# Current Non-Viral-Based Strategies to Manufacture CAR-T Cells

**DOI:** 10.3390/ijms252413685

**Published:** 2024-12-21

**Authors:** Leon Gehrke, Vasco Dos Reis Gonçalves, Dominik Andrae, Tamas Rasko, Patrick Ho, Hermann Einsele, Michael Hudecek, Sabrina R. Friedel

**Affiliations:** 1 Medizinische Klinik und Poliklinik II und Lehrstuhl für Zelluläre Immuntherapie, Universitätsklinikum Würzburg, 97080 Würzburg, Germany; 2 Fraunhofer-Institut für Zelltherapie und Immunologie, Außenstelle Zelluläre Immuntherapie, 97070 Würzburg, Germany

**Keywords:** non-viral, gene transfer, CAR-T cells, transposase, sleeping beauty, piggybac, programmable endonuclease, non-integrating, CRISPR, transient

## Abstract

The successful application of CAR-T cells in the treatment of hematologic malignancies has fundamentally changed cancer therapy. With increasing numbers of registered CAR-T cell clinical trials, efforts are being made to streamline and reduce the costs of CAR-T cell manufacturing while improving their safety. To date, all approved CAR-T cell products have relied on viral-based gene delivery and genomic integration methods. While viral vectors offer high transfection efficiencies, concerns regarding potential malignant transformation coupled with costly and time-consuming vector manufacturing are constant drivers in the search for cheaper, easier-to-use, safer, and more efficient alternatives. In this review, we examine different non-viral gene transfer methods as alternatives for CAR-T cell production, their advantages and disadvantages, and examples of their applications. Transposon-based gene transfer methods lead to stable but non-targeted gene integration, are easy to handle, and achieve high gene transfer rates. Programmable endonucleases allow targeted integration, reducing the potential risk of integration-mediated malignant transformation of CAR-T cells. Non-integrating CAR-encoding vectors avoid this risk completely and achieve only transient CAR expression. With these promising alternative techniques for gene transfer, all avenues are open to fully exploiting the potential of next-generation CAR-T cell therapy and applying it in a wide range of applications.

## 1. Introduction

The successful clinical implementation of chimeric antigen receptor (CAR) T cells depends on the quantity and quality of the drug product, which are strongly affected by the manufacturing process. The critical attributes of CAR-T cell products are safety, purity, consistency, potency, and persistence [1]. There are many ongoing efforts to replace the manual with automated and centralized point-of-care manufacturing processes, as well as a shift from ex vivo production toward the in vivo generation of CAR-T cells. The development and optimization of innovative technologies, reagents, and devices for delivering genes of interest (GOI) aim to transition the production of CAR-T cells even further in the near future.

Despite major research efforts and advances in recent years, all approved CAR-T cell drug products still use the same complex manufacturing workflows generated specifically for each individual patient. Current state-of-the-art CAR-T cell production involves the isolation of peripheral blood mononuclear cells or T cells from leukapheresis products and their subsequent activation. Afterward, the cells are subjected to gene transfer to express the CAR construct and are expanded for several days to achieve the infusion dose [2]. Finally, the T cells are harvested, the cell numbers and CAR expression levels are determined, and the products are filled according to the required dosage. CAR-T cell production is still a lengthy process that is largely carried out manually or semi-automatically in highly specialized GMP facilities, which results in high costs and limits the production throughput. Commercially approved CAR-T cell products currently have price tags of roughly $420,000 to 550,000 per batch [3]. Such high costs are prohibitive to the widespread application of CAR-T cell therapy.

One major cost driver in the clinical production of CAR-T cells is the viral vector or nucleic acid material for gene transfer, which must comply with GMP requirements and fulfill strict quality control specifications. The production of GMP-grade viral vectors is expensive, laborious, and time-consuming and requires S2 laboratories with specially trained personnel. Lot sizes are limited for GMP production, resulting in the need to adjust for lot-to-lot variations during CAR-T cell manufacturing.

All six EMA- and FDA-approved CAR-T cell products rely on viral gene delivery vectors, namely, lentiviral vectors (LVV) or gamma retroviral vectors (γRVV), to achieve gene transfer. Viral vectors are able to introduce payloads of up to 8–10 kb of genetic material into cells. As non-targeted gene transfer systems, lentiviral vectors tend to integrate transgene payloads into actively transcribed genes and transcription start sites, whereas γRVVs frequently integrate into promoter regions, exons, and regulatory elements [4,5]. This is associated with an increased risk of activation of proto-oncogenes or disruption of tumor-suppressor genes, and therefore, the induction of malignant transformation [6,7].

Despite decades of experience with virally produced CAR-T cells, the paradigm is shifting toward the implementation of alternative gene transfer strategies. Besides safety aspects, factors like high production costs, long production times, and regulatory and logistical burdens drive this shift. Transposase systems can achieve high gene transfer rates, while programmable endonucleases can be targeted to specific integration sites, reducing the potential risk of malignant transformation. Non-integrating vectors present opportunities to completely circumvent integrational mutagenesis, and therefore, minimize risks pertaining to the genetic safety of the drug product. Nonetheless, each of these alternative gene transfer systems has limitations that will require further optimization. While the delivery of protein complexes or naked DNA/RNA vectors requires the development of compatible and potent delivery systems able to overcome specific challenges, delivery platforms, like electroporation, cell squeezing, or liponanoparticles, are not the focus of this article and are systematically reviewed elsewhere [8,9].

In general, gene transfer should be efficient and lead to a consistent output. It should not affect the vitality, quality, and fitness of the cells, and of course, be safe to use. In addition, factors such as the availability of GMP quality, price, logistics, and batch-to-batch consistency of starting materials have to be considered.

**Figure 1:**
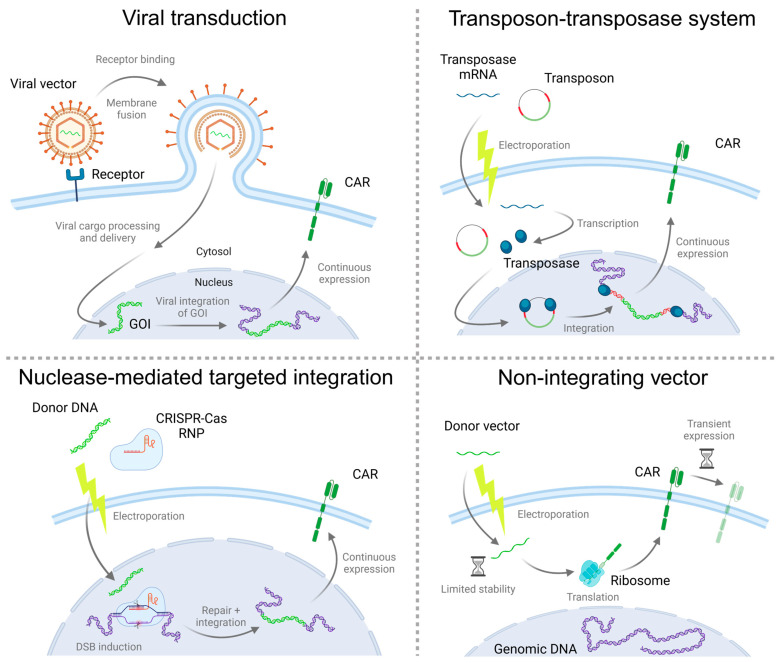
Overview of different strategies for gene transfer. Viruses, transposases, and programmable endonucleases mediate stable integration of the GOI into the genome, and therefore, stable CAR expression. Non-integrating vectors do not induce gene integration and thus induce transient CAR expression as long as the vector is present in the cell. The respective mechanisms and methods of delivery are depicted in a generalized but not necessarily inclusive manner. Transposase protein (blue ellipses); transposon ITRs (red DNA); CAR/GOI (green RNA/DNA/protein); genomic DNA (violet).

In this review, we discuss current strategies for virus-free gene transfer used in the manufacturing process of CAR-T cells (Figure 1), their advantages and disadvantages, and future perspectives of the field (summarized in Table 1).

## 2. Stable Gene Transfer by Transposases

Transposition is a process by which mobile genetic segments, called transposons or transposable elements, change their positions within a genome. Transposons occur naturally in a wide range of organisms, including bacteria, plants, and animals, and constitute more than 45% of the human genome [17]. Transposons can be grouped into either class I, called retrotransposons, or class II transposons, also known as DNA transposons [18,19].

Retrotransposons use a mechanism in which the transposon DNA segment is first transcribed into an mRNA intermediate and then translated back to DNA by reverse transcriptase, which is often coded as part of the transposable element itself. This newly created DNA segment can then be inserted at another location in the genome [18,19].

DNA transposons circumvent the need for an mRNA intermediate or reverse transcriptase using a cut-and-paste mechanism. Here, the transposase enzyme excises transposon DNA segments directly from the donor DNA and reintegrates them at another location within the genome [18,19]. The two most commonly used DNA transposons for the generation of CAR-T cells are Sleeping Beauty (SB) and PiggyBac (PB), both of which achieve stable, non-targeted gene transfer, as it is achieved with viral vectors.

The transposase and the transposon should be encoded on two separate vectors to reduce the risk of transposase integration into the genome [20]. This risk can be further mitigated if transposase is encoded as mRNA or delivered as a protein. The amount and ratio of transposase and transposon vectors can influence gene transfer rates and transgene insertion copy numbers [12,21]. Gene transfer rates and cell viability can be enhanced by replacing conventional plasmids with DNA vectors of reduced size and lacking bacterial backbones, such as minicircles [21,22]. In clinical applications, in particular, it is recommended that the transposase is encoded on mRNA, leading to a transient expression of that enzyme and, therefore, enhancing the safety of the CAR-T cell product [21,23]. This reduces the risks of (1) triggering an immune response against CAR-T cells, (2) integrating transposase DNA into the genome, and (3) persistent transposase expression, which allows subsequent re-transpositions to leave footprints across the genome, thus increasing the risk of mutagenesis [24,25,26]. The transposase- and transposon-encoding vectors are introduced into the target cells using various delivery techniques, such as electroporation, lipofection, or nanoparticles [21,22,27].

### 2.1. Sleeping Beauty

**Figure 2:**
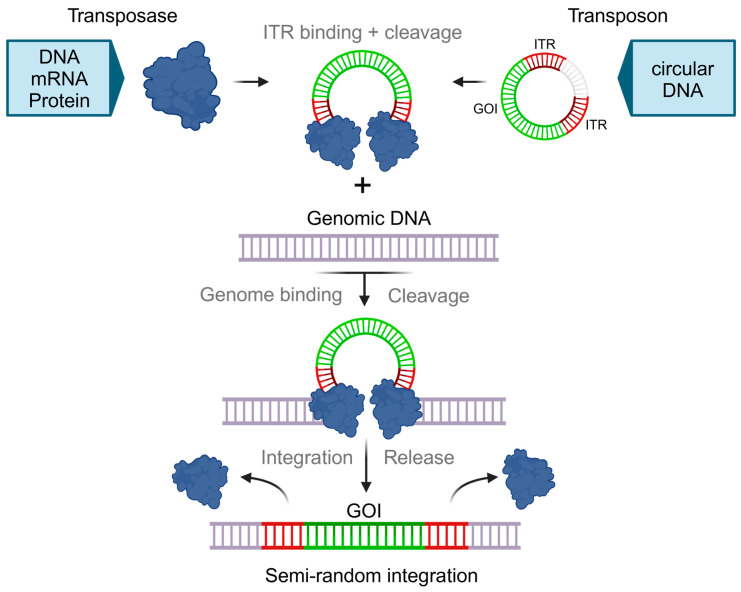
Transposon-based cut-and-paste gene transfer. Transposon and Transposase are encoded separately. The transposase can be delivered as DNA, mRNA, or protein. A transposon carrying the GOI requires delivery as circular DNA. The SB protein binds to the ITR region of the transposon vector and forms a synaptic complex, in which both ends of the transposon are held together and excised from the DNA vector. For SB, the transposon is integrated at a random TA target site in the host cell genome, resulting in stable expression of the GOI. SB protein (blue); transposon ITRs (red); GOI (green).

The class II Tc1/mariner-type transposase Sleeping Beauty (SB) utilizes a cut-and-paste mechanism for gene integration of up to 8 kb in cargo size (Figure 2). The transposon DNA sequence includes the GOI and is flanked on both sides by terminal inverted repeats (TIR) that are 200–250 base pairs in length. Each TIR is bound specifically by two SB transposases forming a homodimer. These homodimers form a paired-end tetrameric complex and excise the transposon from the donor site. The transposases then induce a double-strand break at a random TA position in the genome, at which the excised transposon is integrated [28]. The excision site is repaired by non-homologous end-joining (NHEJ), leaving a CAG footprint [29]. When analyzing the insertion sites within the genome, it was found that SB has a potentially safer, close-to-random integration profile compared to viral vectors or PiggyBac (PB) transposase [4,21,26,30].

The SB transposase, which occurs naturally in teleost fish where it is transpositionally inactive due to the accumulation of mutations, was first described in 1997 by Zoltan Ivics and colleagues. They used synthetic sequence reconstruction to revive the activity of this enzyme [31]. Step-by-step, they predicted the original putative transposase sequence and thereby designed, inter alia, the 340 amino acid protein SB10, which proved to be tranpositionally active in vertebrate cells [31]. Since then, there have been a number of optimizations to further increase the transposition rates when using SB. The transposase itself was optimized through amino acid substitutions [32] and high-throughput screening for hyperactive variants [33], resulting in the development of a stable, highly soluble SB variant that can be delivered in the protein form [30]. The transposon vectors were optimized by adjusting the nucleotide residues within the TIRs sequences [31,34,35,36].

There are many examples of the use of SB in the preclinical production of CAR-T cells. As early as 2008, it was shown that CD19-CAR-T cells could be produced by electroporating freshly isolated, non-activated T cells with the SB transposase variants SB10 or SB11, although gene transfer rates were low, and CAR-expressing cells, therefore, had to be enriched [37,38]. Today, the SB variant SB100x is mainly used for the efficient production of CAR-T cells, CAR natural killer (NK) cells, or CAR cytokine-induced killer (CIK) cells, achieving integration rates of around 50% [21,39], 40% [40], or 60% [41], respectively.

Further, SB was the first transposase used for clinical CAR-T cell manufacturing [42,43]. In 2016, Kabriaei and colleagues published their phase I studies on the treatment of 26 NHL and ALL patients with second-generation, allogeneic, and autologous CD19-CAR-T cells produced by Sleeping Beauty-based gene transfer. In these clinical trials, T cells were electroporated with two plasmids encoding the CAR-transposon and SB11 transposase and subsequently expanded with antigen-presenting feeder cells. Analysis of the cell products revealed no changes in the TCR repertoire, low VCN of 1 (allogeneic) and 1.3 (autologous), and an integration profile that gave no indication of a common insertional hotspot. The mean CAR expression in CD3+ cells was 83% (allogeneic) and 88.5% (autologous). CAR-T cells proved to be safe and could be detected in an average of 51 days (allogeneic) and 201 days (autologous) in peripheral blood [43,44].

SB transposition has additionally been used to produce donor-derived CD19-CAR-expressing CIK cells to treat B-ALL after allogeneic hematopoietic stem cell transplantation. CIK cells are artificially generated CD3+ and CD56+ natural killer T cells. For this purpose, PBMCs were electroporated with plasmids encoding a third-generation CD19-CAR and SB11 transposase and differentiated using IFN-y, IL-2, and CD3 mAbs. CAR-CIK production lasted a total of 20–32 days. The mean CAR expression rate was 43%, the mean transgene copy number per cell was 3.5, and a highly polyclonal repertoire of cells was detected at early time points. Most of the cells produced had a CD8 effector memory phenotype, and 46% were positive for CD56. Robust expansion of CAR-expressing cells was achieved in all patients, and cells could be detected for an average of 94 days [45,46,47].

The CARAMBA clinical trial, in which fresh, autologous SLAMF7-CAR-T cells are used to treat multiple myeloma, was launched in 2021. Here, CD4^+^ and CD8^+^ CAR-T cells are activated separately by CD3/CD28 stimulation and then electroporated with CAR-encoding minicircle DNA and SB100x-encoding mRNA. The production of the cell product takes 14 days, resulting in a vein-to-vein time of 16 days [2].

Further clinical trials based on the SB gene transfer are planned or already ongoing, e.g., one using CD19-CAR-T cells for the treatment of relapsed or refractory B-cell lymphomas (EudraCT 2022-001040-23) and another using ROR1-CAR-T cells for the treatment of hematologic and solid tumors (EudraCT 2022-003728-41).

The application of SB has so far shown great promise, and the full potential of SB gene transfer is yet to be realized in the field of CAR-T cell research. Until now, one of the main shortcomings of the application has been that transposon and transposase are usually introduced into the cells by electroporation—A process that is always accompanied by a drop in cell viability. New methods of gene transport could offer a solution to this problem. However, high gene transfer rates can be achieved with this technique, and SB has been proven to have a more favorable integration profile and lower potential risk of insertional mutagenesis compared to other gene transfer methods [4,43,48,49]. It offers the possibility of one-step modification of CAR-transfer together with other gene engineering techniques, e.g., CRISPR gene editing [50,51]. Furthermore, SB has already been successfully tested in automated manufacturing devices [39], and the production of starting materials is less costly and less complicated compared to that of viral gene transfer.

### 2.2. PiggyBac

PiggyBac (PB) is also a class II transposon that originates from insects and was first described in cabbage looper [52]. It has a size of 594 amino acids and contains DNA-binding domains, a catalytic domain, and dimerization domains [53]. In the transposition process, the PB transposase enzyme binds to the TIR sequences, flanking the transposon, excises the transposon, and integrates it at the TTAA chromosomal sites. PB integration was found to be more frequent near CpG islands, DNAse I hypersensitive sites, and transcriptional starting sites compared to SB integration [54]. Compared to SB, PB has a lower theoretical chance to integrate into genomically safe regions and shows an integration profile resembling that of the MLV retrovirus [4]. PB does not leave a footprint at the excision site and produces no target site duplication [52].

As early as 2010, Manuri and colleagues showed that they could use PB transposase for the production of CD19-specific CAR-T cells. Although the gene transfer rate was still extremely low, the number of CAR-expressing cells could be increased to 50% in 21 days with the help of antigen-presenting feeder cells [55].

The same was true for Nakazawa’s group, which published the PB-based production of HER2-CAR-T cells one year later. After electroporation of PBMCs with PB transposon and transposase vectors, the observed gene transfer rates were low, but CAR-expressing T cells could be enriched to around 40% by magnetic selection and expansion [56].

Efforts to enable the clinical use of PB-generated CAR-T cells have gained traction [57,58], and the transposition process has been steadily optimized by developing hyperactive PB mutants, reducing the lengths of TIR sequences, and replacing conventional plasmids with smaller DNA vectors [59,60,61].

However, this development slowed in 2021 with the publication by Micklethwaite and colleagues, who reported the appearance of malignant CAR-T cells in the first-in-human study involving CD19-CAR-T cells produced by PB transposition. In this study, known as the CAR-TELL trial, CD19-CAR-T cells were used to treat B-cell malignancies after allogeneic hematopoietic stem cell transfer (HSCT). For this purpose, the CAR-transposon plasmid and PB mRNA were electroporated into T cells from the allogeneic HSCT donor, achieving high gene transfer rates of around 72%. The release tests for unwanted autonomous T cell growth remained unremarkable. Nevertheless, two of the 10 treated patients developed CAR-positive T cell lymphomas. The first patient had received three doses of CAR-T cells from his HLA-matched sibling donor and developed T cell lymphoma of the effector T helper type 1 subtype. The malignant cells had a relatively high transgene copy number (24), but this was not increased compared to non-malignant cells of the same product. Whole genome sequencing of the monoclonal cells revealed insertions within genes and within 2.5 kb of the transcription starting site, but no insertion into commonly known oncogenes. Transcriptome analysis showed transgene promoter-driven upregulation of sequences at the insertion sites and indicated transcriptional read-through processes of up to 1000 kb. These read-through events included non-coding intronic and out-of-frame sequences, as well as in-frame exon expression of four genes. In addition, widespread copy number gains and losses were observed in tumor-suppressor genes and oncogenes. The second patient exhibited monoclonal CAR-expressing CD8^+^ T cells. The transgene copy number was only four, but read-through events were again detectable. The cells of both patients had insertions in the intron of the BACH2 gene, which led to its reduced expression. Since there were some abnormalities but no obvious known trigger described, the cause of the transformation could not be clearly determined [62,63].

Until these triggers have been clearly identified and a strategy to prevent malignant transformation has been developed, research on PB-generated CAR-T cells will certainly continue with the handbrake on.

### 2.3. Other Transposases

Two other transposases that have been used in the context of CAR-T cell manufacturing are transposable element of Oryzias latipes, number 2 (Tol2) [64] originating from medakafish [65] and TcBuster [66], which was originally isolated from the red flour beetle [67].

Different isoforms of Tol2 exist with different levels of activity [68]. The transposition efficiency of Tol2 is increased by codon optimization and shortening of the transposable element [69]. At 10–11 kb, the cargo capacity of Tol2 is high enough for comprehensive T cell modification [69,70]. Tol2 preferentially integrates at AT-rich palindrome-like DNA regions and in transcription units, mostly in introns [71]. It generates DNA duplications of eight base pairs and leaves footprints after excision [72].

TcBuster can transfer large cargo with sizes of 10–12 kb. A hyperactive mutant was generated using high-throughput screening of 3 million variants, significantly increasing gene transfer rates in human NK and T cells [66]. Integration site profiling revealed a safer integration profile compared to lentiviral vectors but a greater tendency to integrate into transcripts, exons, transcription start sites, CpG islands, and DNAse I cleavage sites compared to SB [66,73]. TcBuster induces eight base pair target site duplications, inducing footprints at excision sites [67].

## 3. Targeted Integration Using Programmable Endonucleases

**Figure 3:**
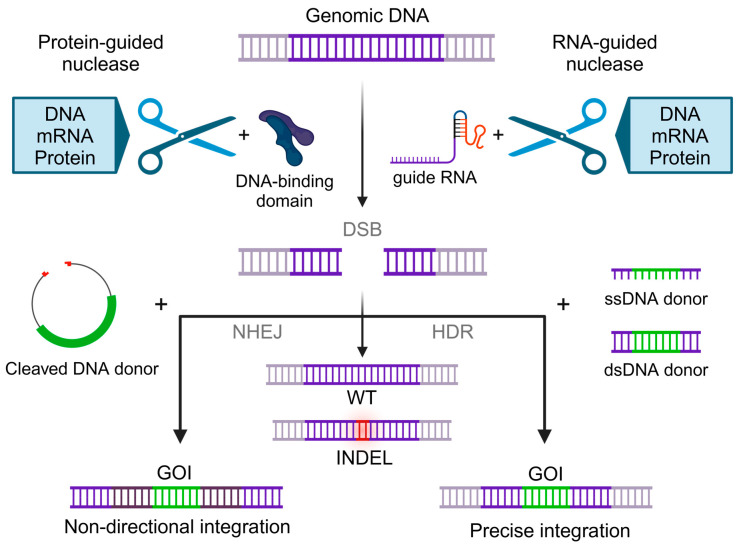
Targeted transgene integration using double-strand break induction via programmable nucleases: Genomic DNA containing the targeted sequence is cleaved by protein-DNA interactions or RNA-guided endonucleases. The resulting double-strand break (DSB) is repaired either by the error-prone non-homologous end-joining (NHEJ) pathway or by homology-directed repair (HDR). This results in correct repair or insertions and deletions (INDELs). Supplying a single- or double-stranded DNA donor template carrying homologous sequences can facilitate precise integration of the GOI at the target locus. Concurrent delivery and cleavage of a non-homologue DNA donor template can facilitate non-directional targeted integration. Targeted genomic DNA sequence (violet); GOI (green); INDEL (red with halo).

Targeted integration of GOI can be achieved by co-delivery of programmable endonucleases, which induce double-strand breaks (DSBs) at selected sites in the genome, along with transgenes encoded within DNA repair template vectors (Figure 3). The key component is a programmable nuclease, which relies on RNA molecules or DNA-binding protein domains to recognize and cut the targeted genome site. The characteristics of the supplied DNA donor template help to instruct the mechanism of repair, thus ensuring the precision of the integration.

### 3.1. Programmable Endonucleases: RNA-Guided Nucleases

The discovery of the Clustered regularly interspaced short palindromic repeats associated with the Cas endonucleases (CRISPR-Cas) system has revolutionized the field of gene therapy due to its unparalleled versatility, cost-effectiveness, and ease of use. Engineered CRISPR-Cas systems, most prominently implementing variants of CRISPR-associated (Cas) nuclease 9, offer a highly flexible two-component system combining an endonuclease and an easy-to-design short guide RNA (sgRNA) for selective targeting. The sgRNA mediates DNA-binding via Watson-Crick base-pairing to instruct the specific targeting of virtually any defined sequence within the genome. The only prerequisite is the presence of the protospacer-adjacent motif (PAM), a three-nucleotide sequence (NGG) immediately adjacent to the freely selectable, 20 nucleotide (nt) long recognition sequence that directs binding to the specified target locus. Incremental improvements to the Cas9 endonuclease have allowed for the generation of higher specificity or higher fidelity variants like the aptly named Cas9 Sniper [74] and Cas9HiFi [75], respectively, effectively expanding the CRISPR/Cas9 toolbox. Additionally, other engineered variants offer solutions to broaden the range of genomic sequences potentially targetable by Cas9 by relaxing the sequence restrictions (Cas9 SpG, NGN) or almost entirely removing PAM requirements (Cas9 SpRY, NRN/NYN) [76]. Notably, Cas9 was used for the original study, which showed a CAR sequence being inserted within the T cell receptor alpha constant (TRAC) gene to generate TCR-deficient knock-in CAR-T cells [77].

Other RNA-guided nucleases in and outside the Cas family have expanded the available toolset. The most prominent RNA-guided agent, aside from Cas9, is Cas12/CPF1 and its variants [78,79]. The combination of the two systems enables multiplexed orthogonal editing without guide switching. Multiplexed transgene delivery has already been demonstrated, generating multiplex-edited T cells expressing CAR within the TRAC locus and silencing human leukocyte antigen HLA I and HLA II expression [80].

### 3.2. Programmable Endonucleases: Protein-DNA Interaction-Guided Nucleases

Zinc-finger Nucleases (ZFNs), Transcription Activator-Like Effector Nucleases (TALENs), and Meganucleases are the most prominent programmable nucleases that use protein-based recognition of DNA motifs to mediate specificity. The protein domain integrity necessary for correct folding and subsequent DNA binding of these nucleases limits the number of potentially targetable sequences in the genome, but the long history of usage provides an advanced understanding of targeting mechanisms and off-target effects.

Meganucleases are naturally occurring monomeric endonucleases that are able to specifically recognize long DNA sequences (>18 nt) and mediate cleavage. Accordingly, retargeting requires work-intensive combinatorial re-engineering of the complete protein and can be restricted to a limited set of targetable sequences.

ZFNs and TALENs are recombinant nucleases that use DNA-binding protein domains in combination with a FokI Nuclease Domain. Both ZFNs and TALENs are designed as monomeric proteins that cut one strand of DNA and act in dimers to induce DSBs. To achieve the juxtaposed orientation necessary to generate a DSB at the same position, each pair of TALENs or ZFNs requires two distinct target sequences, and DSB induction requires successful dimerization subsequent to the independent recognition of both sequences. This enables well-designed ZFN and TALENs to cut DNA with high specificity and reduce the risk of off-target effects. ZFN derive their specificity from combinations of zinc-finger (ZF) domains that recognize three bases each. State-of-the-art ZFN specificity is conveyed by the specific recognition of at least 18 nucleotides. The retargeting of ZFNs is a time-consuming engineering process that is further complicated by non-targetable nucleotide triplet combinations, reciprocal influences between single ZF domains, and cytotoxicity [81].

For TALENs, DNA binding is mediated by combining different repeat-variable di-residues (RVDs) polymorphic positions in highly conserved amino acid repeats that specifically recognize and bind one nucleotide [82]. The combination of RVDs matching the four DNA bases creates DNA-binding domains that recognize 18–20 nt, lifting target sequence restrictions and allowing for comparatively easy retargeting [83]. While all nucleases are predominantly used for combinatorial editing in combination with other strategies, including viruses for CAR delivery, protein-DNA targeting has proven to be a flexible and effective technology, even for sophisticated CAR-T cell engineering approaches, including targeted CAR integration [84,85].

### 3.3. Transgene Integration via Cellular DSB-Repair Mechanisms

Innate eukaryotic surveillance and repair mechanisms aim to maintain genomic stability and sequence integrity by preventing long-lasting DNA damage [86]. Targeted transgene integration can be achieved by hijacking endogenous cellular DNA repair mechanisms that are activated upon the occurrence of a double-strand break.

Repair pathways can either be homology-dependent or-independent, with the choice and outcome of the repair mechanism being a highly regulated, multifactorial process instructed via DNA end configuration, sequence identity, spatial alignment, and input from several central pathways regulating the cell cycle or homeostasis. Both the most prominent somatic DNA damage repair pathways, non-homologous end-joining (NHEJ) and homology-directed repair (HDR), can be exploited to integrate transgenes into open break sites [87].

HDR mechanisms are potentially error-free multi-step repair processes that allow for template-based repair, fixing the DSB via templated synthesis of a complementary DNA strand and subsequent recombination [88]. In somatic cells, synthesis-dependent strand annealing (SDSA), which results in conservative repair without crossover, is the predominant HDR repair pathway and is active during the S and G2 phases of the cell cycle. Substitute pathways include single-strand annealing (SSA) or alternative end-joining by microhomology-mediated template switching (MMEJ) [89]. By designing DNA donor vectors to flank the transgene with homology arms matching the sequences upstream and downstream of the DSB, the endogenous repair machinery can facilitate precise transgene integration using endogenous HDR pathways. The single-strand or double-strand DNA donor template carrying the transgene can vary in size and end-modification, with efficient integration of several kb long constructs (e.g., CARs) being the state-of-the-art [77]. Building on homology repair, short single-stranded templates can be used to introduce single nucleotide changes or corrections of short stretches of up to 50 base pairs. This strategy has been exploited to facilitate the integration of larger transgenes, such as CARs, via primed microhomologues-assisted integration (PAINT) [90,91].

In contrast, NHEJ is a rapid, high-capacity repair pathway that requires no or minimal template sequence for reference and acts independently of the cell cycle state on open DNA ends, representing the default repair mechanism upon spontaneous DSBs [92]. This repair pathway joins open DNA ends in an error-prone manner, resulting in the resolution of DSBs with insertions or deletions (INDELs) in approximately 70% of repaired double-stranded DNA [93]. NHEJ is often taken advantage of to induce INDELs with programmable endonucleases to knock out relevant genes by disrupting the endogenously encoded genetic information. It can further be used to facilitate homology-independent targeted integration (HITI) of transgenes via concurrent donor and genomic target DNA cleavage in a site-specific and efficient, albeit non-directional, INDEL-prone, and less precise manner [94].

The limiting factors for efficient targeted integration approaches using HDR are usually attributed to DNA-induced toxicities, which increase with the total DNA amount and length of the transgene, and the relatively low natural HDR frequencies, which are insufficient for cell product manufacturing [95]. Recent developments aim to increase the efficiency of HDR-mediated transgene integration by (1) synchronizing cell cycle states prior to gene editing [96], (2) modifying the HDR template to either facilitate interaction with HDR-related proteins [97], or (3) hiding the template DNA from triggering innate immune pathways that sense and respond to foreign DNA, thus reducing the cytotoxicity experienced by target cells during gene delivery. The state-of-the-art strategy to increase HDR-mediated donor integration is to administer small molecules that inhibit key factors in the NHEJ pathway, such as the DNA-dependent phosphokinase catalytic subunit (DNA-PKc), which is rapidly recruited to the DSB ends and facilitates a crucial, deterministic step to initiate NHEJ repair. In this way, the DNA-PKc–treated cells are forced to rely on the remaining HDR-mediated repair pathways or otherwise suffer apoptosis due to genotoxicity [98,99,100].

### 3.4. Strategic Application and Risk Mitigation

Targeted integration allows for loci-specific delivery of transgenes, making loci-effects, including 3D chromatin structure, chromatin accessibility, and transgene copy number predictable and selectable. Several loci are considered to be “safe harbor” sites, such as adeno-associated virus site 1 (AAVS1), protein phosphatase 1 regulatory subunit 12 C (PPP1R12C), or chemokine receptor 5 (CCR5), all of which have been shown to be clinically safe loci for targeted transgene integration. The specific disruption of these loci has not revealed any detrimental impact on cell differentiation or function, thus ensuring that integration at these sites has no relevant negative effects on the potency of the edited cells [101].

Further, the simultaneous disruption of targeted loci upon (CAR) transgene integration can even be advantageous for certain cell engineering approaches. T cell receptor (TCR)-KO CAR-T cells mitigating graft-versus-host disease have proven useful for non-targeted CAR delivery and approaches using concurrent CAR integration into the TCR-alpha-chain locus [102]. Concurrent KO of safe-harbor locus CCR5 during CAR integration yields HIV-resistant CAR-T cells [103], and KO of immune checkpoint receptors allows for the generation of CAR-T cells that are not impeded by inhibitory signals within suppressive tumor micro environments [103]. Combinatorial approaches can be used to eliminate HLA expression, relieving the requirement for donor HLA matching. These multi-target editing strategies can be employed in combination with different modes of CAR delivery to generate allogeneic “universal” or “of-the-shelf” CAR-T cells [104]. Linking CAR expression to TCR signaling via site-specific integration of CARs into the human TRAC locus has been associated with activation-induced downregulation of CAR expression, improved therapy outcome, and reduced exhaustion of T cells after antigen encounter [105]. The potential of such sophisticated strategies is illustrated by the engineering of receptors that directly incorporate the desired endogenous protein parts via integration at the endogenous locus. Prominently, the CD247-domain-deficient CAR, integrated into the CD247 locus, allows for the generation of CAR-T and CAR-NK cells, that express the CAR under control of the endogenous promoter of CD247 and incorporate the endogenously encoded signaling domain into the final surface protein [106]. Contrary to TRAC-KI approaches, this strategy enables the generation of CAR-NK cells expressing CAR under an endogenous promoter active in NK cells and simultaneously reduces the cargo size upon donor delivery. Intricate engineering approaches at this level have also been used to create new engineering-based receptor classes like HLA-independent T cell (HIT) receptors [106,107].

Although site-specific genome-editing technologies have enabled cell engineering with unprecedented precision, the applications to CAR-T cell manufacturing continue to face significant challenges in feasibility and safety. The low scalability and inefficiency of gene delivery pose problems for cell products that require large quantities of genome-editing material. Additionally, detrimental effects resulting from genetic editing (or editing-related processes) raise genomic safety concerns [95]. Off-target DNA cleavage can disrupt unrelated genes or even lead to larger translocations [108]. Several genome-wide technologies have been developed to predict and detect intended and unintended editing outcomes for in silico, in vitro, and in vivo applications. Guide and Circle Seq are the most prominent tools for detecting off-target aberrations [109,110]. CAST-Seq focuses on large translocations [111] recently employed to re-evaluate the safety profiles of base editors and TALENs [112]. The recently presented MEGA dPCR relies on digital droplet PCR (ddPCR) and aims to integrate most on- and off-target assessments into one assay [113]. Currently, a diverse assortment of methods needs to be applied to estimate and subsequently mitigate the genomic effects of nucleases. Furthermore, the real-world frequency of these genomic abberations, as well as their relevance (e.g., transformative mutations), remains hard to assess.

Due to the risk of off-target editing and imperfect in silico prediction algorithms, it is necessary to make an individual risk assessment for each new target and each new CRISPR-guide that is used for the generation of CAR-T cells. Currently, the need to extensively evaluate the safety, efficacy, and individual off-target effects has resulted in new clinical trials for products with minor changes in the targeting agent [114,115] (see FDA guidance for industry on human gene therapy products incorporating human genome editing from January 2024).

Another significant challenge to the widespread application of CAR-T cells generated by targeted integration is the current limitation of DNA donor templates. The need for DNA donors excludes many modern gene editing delivery approaches that rely on delivering all used agents, either as less immunogenic RNA or directly as proteins. This largely restricts the truly virus-free application of ex vivo approaches for delivering agents via nucleofection. In clinical applications, delivery via non-integrating AAV Vectors is usually needed to facilitate high editing efficiency and allow for simultaneous delivery of the DNA donor template, making it a not truly virus-free strategy and raising the respective costs of the therapies due to expensive vector production. Novel strategies to overcome this hurdle are being developed [94].

The restrictions imposed by safety requirements and transgene delivery options make CAR-T cells generated by targeted insertion best suited for the exogenous production of allogeneic T cells. In this setting, multiplexed, targeted genome editing facilitates the development of universal or off-the-shelf CAR-T cells via the simultaneous disruption of genes involved in HLA-mismatch–induced rejection. Allogenic CAR-T cell therapies hold great translational promise as off-the-shelf products. However, while gene-edited CAR-T cells are already in clinical use, there have been few trials of CAR-T cells using gene editing for CAR delivery [14].

## 4. Gene Transfer by Non-Integrating Vectors

A promising alternative for gene delivery in CAR-T cell immunotherapy is the use of non-integrating strategies. DNA- or mRNA-based vectors can deliver GOIs without requiring the co-delivery of any genomic editing machinery and have the potential to reduce both the cost and length of manufacturing protocols while bypassing the risks of insertional oncogenesis, genomic translocations, and rearrangements [116,117]. To date, the major challenge for these vectors stems from the transient nature of gene transfer to engineered cells. However, with different half-lives, transformation with both DNA and mRNA results in transient transgene expression, effectively abolishing the capacity for long-term CAR-T cell expansion and persistence. Advancements in the design and structure of both DNA and RNA vectors that enable improved persistence or even vector self-replication have the potential to circumvent these aspects and renew interest in such technologies.

### 4.1. DNA-Based Vectors

Bacterial-derived plasmids have historically been the most frequently used DNA-based vectors in cancer gene therapy. Their easy design, coupled with low cost of manufacturing, high stability, and long shelf life, are the advantages that make these strategies attractive. The advent of minicircles and nanoplasmids, which greatly reduced the vector backbone size (from the traditional 2 kb observed in plasmids to below 500 bps) and the amount of bacterial-derived sequences, further addressed challenges like bacterial backbone-induced GOI silencing, toxicity to target cells, and cellular and metabolic disturbance. These improvements are especially important in the context of immunotherapy. T cells are characterized by their extreme sensitivity to immunogenic exogenous DNA, which induces rapid functional loss or even apoptosis [118]. Electroporation with DNA minicircles has been shown to reduce gene transfer-associated toxicity while simultaneously resulting in higher transfection efficiencies when compared to conventional plasmids.

However, due to the need for an in vivo site-specific recombination step coupled with chromatography-based purification, minicircle production has a low scalability potential characterized by low manufacturing yields. Nanoplasmids, which implement a ~500 bp RNA-out selection marker [119,120], offer the same transfection benefits as minicircles while implementing different manufacturing setups [121].

Continuous, systematic refinement of DNA vector composition has allowed for the development of non-integrating lentiviral-derived vectors (NILV) [122] capable of nuclear extra-chromosomal replication. Such constructs make use of the scaffold/matrix attachment region (S/MAR) elements [123], effectively allowing for the generation of persistently modified host cells [16,124]. CD19-CAR-T cells engineered with these NILV-S/MAR vectors display similar levels of CAR expression and on-target in vivo cytotoxic capacity and expansion when compared to LV-engineered T cells [125]. Importantly, the insertion of NILV-S/MAR vectors into the genome of host cells was below the limit of detection.

More recently, Bozza and colleagues further refined this technology by developing the minimally sized nS/MARt DNA nanovector, characterized by its low toxicity when used for the transfection of T cells [126]. nS/MARt DNA nanovectors contain no viral components and have been used for the successful generation of in vitro and in vivo functional CAR-T cells [118].

### 4.2. mRNA-Based Vectors

mRNA-based therapies have ushered in a new era in modern medicine and play a substantial role in the current immunotherapy clinical trial landscape [127,128]. Since mRNA’s main functions are performed in the cytoplasm, effectively bypassing the need to be shuttled to the nucleus, RNA-based therapies offer advantages over DNA-based ones. High transfection efficiencies and low toxicities observed with mRNA-based vectors have refreshed interest in ultra-fast manufacturing protocols. Most strikingly, efficient delivery of mRNA can be achieved by milder delivery methods like cell squeezing technology or liponanoparticles (LNP), allowing for increased viability of the cell product. Such technologies are typically not efficient for DNA delivery, specifically due to the lack of nuclear delivery capacity. Additionally, mRNA production is more convenient, rapid and economical when compared to DNA synthesis. Currently, in vitro transcription (IVT) reactions are preferred over direct chemical synthesis, allowing for higher fidelity in the production of longer mRNA sequences coupled with higher yields of the mRNA product [129,130]. Since IVT reactions result in a mixture of both single- and double-stranded mRNA, purification steps are necessary to reduce the immunogenicity of the mRNA product, as double-stranded RNA is a potent activator of cytosolic innate immune sensors [131].

Recent developments in the mRNA field have allowed for systematic and essential enhancements regarding (a) increased stability in the form of 5′ and 3′ untranslated region optimization [132,133], (b) increased translatability by the incorporation of synthetic caps [134,135,136], poly A tail length [137,138,139], as well as codon and secondary structure optimization [130,140,141], and (c) decreased immunogenicity by the incorporation of alternative nucleotides like pseudouridine [142,143].

Nonetheless, mRNA molecules are short-lived and result in transient expression of the GOI. In clinical trials, CAR transcript detection by post-infusion quantitative PCR demonstrated a rapid decline in GOI expression, with undetectable levels reached after 1–3 days [144,145]. Although the inherent kinetics of mRNA can be advantageous for exploring targets with a high risk of toxicity, it can also make the generation of several doses per patient a logistically and commercially prohibitive process [144]. A clinical trial with mRNA-delivered CD123-CAR-T cells reported failure to manufacture 40% of its planned doses and was eventually terminated [146]. With some patients needing up to six infusions of 1E8 CAR-T cells, as reported in some clinical trials [145,147], failure to generate a drug product can cap the feasibility and commercialization of therapy. Additionally, mRNA is inherently less stable than DNA-based vectors, which means that each additional drug dose correlates with additional costs related to GMP production, drug product storage, and eventual distribution.

Additionally, in another mRNA CAR-T cell trial, frequent administration of the drug product led to an adverse anaphylactic reaction in one of the three patients treated [148,149]. Although no other mRNA-based CAR-T cell clinical trial has reported anaphylaxis, care should be taken regarding the burden of repeated infusions to the patient.

New emerging technologies, such as mRNA delivery systems for sustained release [150,151], self-amplifying RNAs [152], and circular RNA [153], all aim to overcome transient GOI expression issues but have yet to reach clinical trials [154,155].

## 5. Future Perspectives

Four decades after the first antibody/TCR chimeric molecules were first described [156,157], six CAR-T cell products have reached the market, with many more being tested for their safety and efficacy in a wide spectrum of disease types. The therapeutic results observed against hematologic malignancies confirm the potential of CAR-T cells in the field of immunotherapy. To date, all approved products are manufactured by viral gene transfer, although non-viral techniques represent a promising alternative for CAR-T cell manufacturing in the future.

It is important to keep in mind that one of the most decisive factors that determine the suitability of a gene transfer method is its safety regarding the potential to induce genotoxicity. Overall, the risk of secondary T cell malignancies occurring after CAR-T cell therapy is rather low [158], and the mechanisms leading to it, or whether they are induced by CAR gene transfer, are still unknown [62,159]. However, there is at least a potential risk of genotoxicity when using non-targeted integrating vectors. Gene destruction or dysregulation can be caused by transgene integration into active host genes or their regulatory elements. Furthermore, promoters within the transgene itself can alter the expression of genes near the integration site. If this occurs, especially within proto-oncogenes or tumor-suppressor genes, malignant transformation of CAR-T cells could be observed [63,160].

Although not a targeted integration method, SB transposition has been shown to have a safer, and therefore more preferential, integration profile compared to viral vectors or PB transposition in terms of targeted transcriptional units [4,21]. Additionally, transposon-based gene transfer is a cost-effective and easy-to-handle method for producing CAR-T cells. The production of starting materials (DNA and mRNA vectors) that meet clinical quality standards is easily scalable and subject to little variation compared with the production of viral vectors. Gene transfer rates and cell viability have been further optimized, especially by replacing common plasmid vectors with smaller DNA alternatives, such as minicircles [21], allowing for efficiencies comparable to those of viral vectors. The risks of re-mobilization, integration of the transposase expression cassette, and induction of immunogenicity can be reduced by the use of mRNA encoding the transposase [21].

A promising technical improvement to achieve stable therapeutic transgene genomic integration and expression is the combination of a non-integrating adeno-associated virus (AAV) and the Tc1/mariner transposon system. This has resulted in hybrid vectors that exploit the highly efficient targeting properties of the virus through infection and harness stable transposase-mediated gene integration of the transgene in the host cell. These hybrid systems achieved a high rate of stable gene transfer into host cells and, in the case of Sleeping Beauty, possessed a similar safe gene integration profile as previously verified in different mammalian cell lines [161,162,163,164,165].

RNA-based retroelements, which are class I transposons, could be a future alternative. In 2024, Zhang et al. described the approach of precise RNA-mediated insertion of transgenes (PRINT), in which mRNA vectors are combined with sequence-specific integration. Using this technique, transgenes can be directly synthesized by reverse transcription into the genome at a multicopy safe-harbor locus, inducing stable and safe integration [166].

Precision editing, enabled by diverse nuclease technologies and targeted integration approaches, has allowed researchers to engineer cells with unprecedented levels of customization and sophistication. However, although targeted integration approaches using programmable nucleases circumvent the insertional mutagenesis risks associated with (semi-) random integrating vectors, this class of proteins comes with other aspects of genotoxicity that can be attributed to its nuclease activity. Screening technology is rapidly developing to meet the field’s need for genotoxicity and risk assessments. Simultaneously, ongoing advances in nuclease and editing technology are rapidly improving off-target and on-target performances. For example, the development of new techniques, such as base-editing or prime editing, which enable precise nucleotide substitutions, has the potential to mediate edits with even higher precision, and the latter has already been proven capable of safe and targeted introduction of larger transgenes [167,168].

The integration efficiency of current technologies has continuously improved, reaching levels comparable to those of other methods. Nonetheless, the requirement for DNA donors and the respective limitations in the choice of delivery strategy currently exclude efficient in vivo as well as truly virus-free, large-scale manufacturing approaches. Future solutions may allow for enrichment and reduced genotoxicity or altogether eliminate the need for viral delivery, thus sidestepping the costs related to GMP virus production [94]. Until then, CAR-T cells generated using nuclease-mediated targeted integration are best suited for approaches aiming to generate off-the-shelf or universal CAR-T cells where the product can be subjected to intense engineering and rigorous safety screening prior to administration without time constraints [169].

While the temporary expression of genome-manipulating proteins can limit undesirable toxicities, effectively improving the safety profile of these gene editing systems [2,170], mRNA can be used for the transient delivery and expression of the CAR molecule itself [66]. Paired with the lack of genomic integration, high transfection efficiencies, and affordable costs, mRNA vectors offer improved safety profiles that match well with the interest in in vivo generation of CAR-T cells.

Regardless of the non-viral gene transfer system used, directed delivery strategies need to be refined for safe and efficient in vitro or in vivo gene transfer. While gene delivery via viral gene transfer is achieved by natural cellular mechanisms, non-viral gene transfer success is dependent on the development and advancement of concomitant delivery techniques. Until now, most laboratories have used electroporation for the ex vivo transfer of mRNA, DNA, or proteins into cells, a technique that always requires a compromise between a high gene transfer rate and cell viability. Nanoparticles, like LNPs, could be an attractive alternative for the gene delivery of non-viral vectors. These diverse biomaterials offer improved stability of reagents with the option to encapsulate not only proteins but also DNA- or RNA-based vectors, with the possibility of targeting specific cell populations [171]. LNPs have consistently demonstrated their applicability not only in the context of CRISPR-Cas9, emphasizing their potential for the delivery of nucleic acids in immunotherapies [94,172], but also in mRNA vaccines by protecting mRNA from nuclease degradation [173]. In the future, T cell-targeted, antibody-functionalized nanoparticles [172] could be used for the in vivo generation of CAR-T cells, completely bypassing the very expensive and cumbersome ex vivo production in a GMP laboratory.

Overall, the method of gene transfer influences the effectiveness, safety, and accessibility of cell products. It must meet high standards in terms of safety, efficiency, scalability, availability, handling, and cost, among others. It is, therefore, of great importance to further develop and optimize alternative techniques and to regularly weigh their advantages and disadvantages against prevalent virus-based gene transfer methods.

## Figures and Tables

**Table 1:** Comparison of different gene transfer methods.

	Virus	Transposase	Programmable Endonuclease	Non-Integrating Vectors
Delivery Efficiency	highly efficient (can also be used for the delivery of other described systems) restricted by tropism	intermediate (depends on the transfection method and vector size)	intermediate (depends on the transfection method and vector size)	highly efficient mRNA is more efficient than DNA
Cargo Size	depends on the virus (up to 7 kb with lentiviruses, 36 kb with adenoviruses [10])	depends on the transposase (up to 100 kb [11])	single nucleotides to multiple kb	no packaging limitation delivery efficiency decreases with increasing construct size
Expression Persistence	stable expression undirected genomic integration prone to integrate close to transcriptional active sites	stable expression undirected genomic integration integration pattern depends on the transposase	stable expression directed integration based on NHEJ	mainly transient expression ^1^
Copy Number Control	steerable by titrating virus amounts	steerable by transposase activity and transposon quantity [12]	tightly controllable by targeting mechanism	n. a.
Cellular and Genomic Safety	low risk of intracellular detection by STING and TLR low risk of viral particles being detected by the immune system potential risk of insertional mutagenesis	intracellular detection depends on the encoded cDNA low immunogenicity potential risk of insertional mutagenesis depends on the transposase	intracellular detection depends on the encoded cDNA pre-existing immunity toward Cas9 in a small percentage of the human population [13] risk of chromosomal rearrangement and off-target effects	intracellular detection depends on encoded cDNA low immunogenicity no risk of genotoxicity when using mRNA almost no risk of genotoxicity when using DNA
Cost	high	intermediate	intermediate	low
Clinical Translation	several clinical products approved	well represented in clinical trials	currently used in one clinical trial [14]	well represented in clinical trials

^1^ Very few spontaneous integrations are possible from DNA vectors [15]. Episomal proliferation is possible in the presence of specific sequences [16].
